# Probabilistic inference of synaptic dynamics in neocortical microcircuits

**DOI:** 10.1186/1471-2202-14-S1-P403

**Published:** 2013-07-08

**Authors:** Rui P Costa, P Jesper Sjöström, Mark CW van Rossum

**Affiliations:** 1 Neuroinformatics and Computational Neuroscience Doctoral Training Centre, University of Edinburgh, Edinburgh, EH8 9AB, UK; 2 Institute for Adaptive and Neural Computation, School of Informatics, University of Edinburgh, Edinburgh, EH8 9AB, UK; 3 The Research Institute of the McGill University Health Centre, Department of Neurology and Neurosurgery, McGill University, Montreal, H3G 1A4, Canada

## 

**Figure 1:**
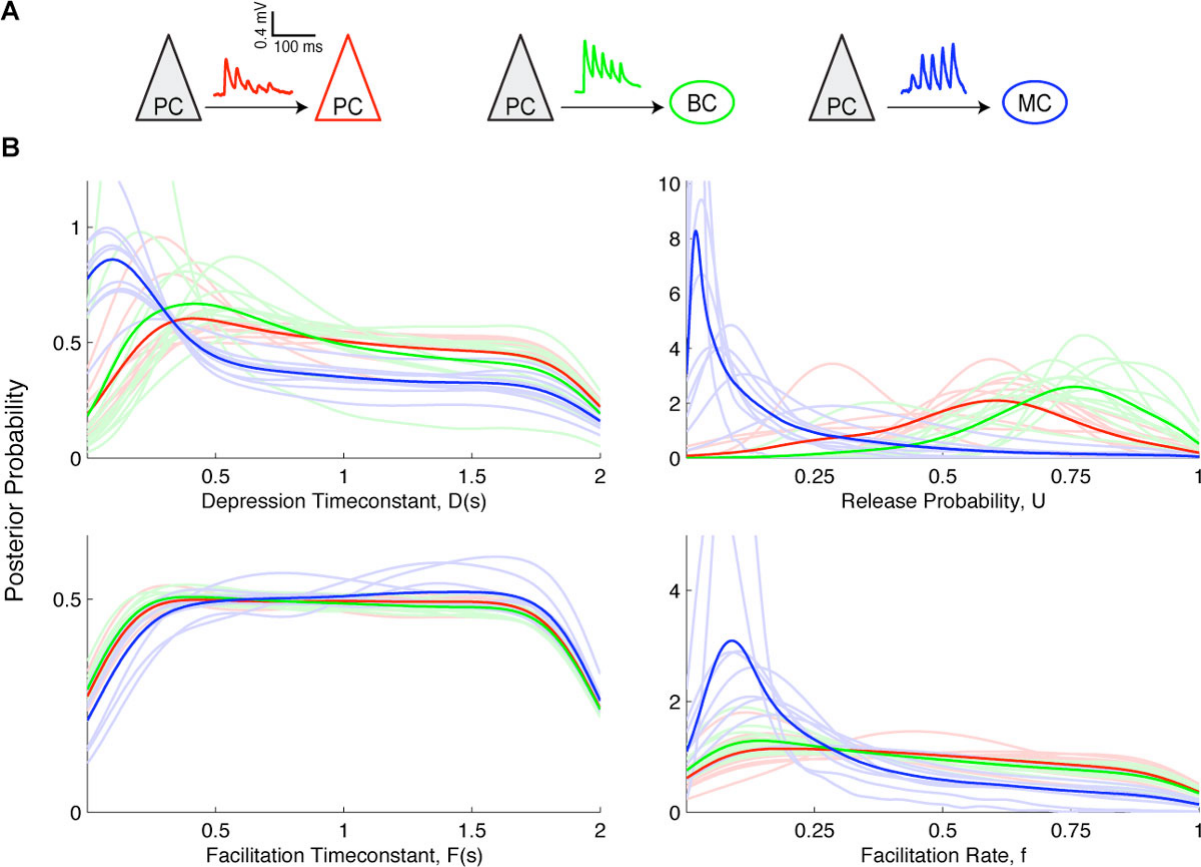
**Posterior distributions of STP parameters from experimental data from visual cortex layer-5**. (A) Sample experimental STP traces are shown for PC-PC (red), PC-BC (green), and for PC-MC (blue) connections. (B) Marginalized posterior distributions obtained using slice sampling from the three different excitatory connections show connection-specific distributions. Light colored lines show individual distributions, while dark colored lines correspond to their average.

Short-term synaptic plasticity (STP) is highly varied across brain area, cortical layer, cell type, and developmental stage (Reyes & Sakmann 1999). This variability is probably not coincidental and since synaptic dynamics shape neural computations, it suggests an important role of STP in neural information processing (Abbott & Regehr 2004). Therefore, an accurate description of STP is a key step towards a comprehensive understanding of neural systems. Many phenomenological STP models have been developed (Markram et al. 1998), but they have typically been fitted to experimental data using least-mean-square methods. With the Tsodyks-Markram model, we find that for typical synaptic dynamics such fitting procedures may give erratic outcomes. A Bayesian formulation based on a Markov Chain Monte Carlo method was introduced as a solution. This formulation provides the posterior distribution over the model parameters given the data statistics. We discovered that standard STP electrophysiology protocols yielded wide distributions over some model parameters. Based on this result we propose experimental protocols to more accurately determine model parameters. Next, the model parameters were inferred using experimental data from three different neocortical excitatory connection types: Pyramidal Cell-Pyramidal Cell (PC-PC), Pyramidal Cell-Basket Cell (PC-BC) and Pyramidal Cell-Martinotti Cell (PC-MC), (see Figure 1). This disclosed connection-specific distributions, which we used to classify synapses. This approach to determining connection-specific synaptic dynamics provides a more comprehensive representation of STP and unveils novel features from existing data.
